# Expedited approval of cancer drugs without randomized controlled trials: Too good to be true?

**DOI:** 10.18632/oncotarget.25799

**Published:** 2018-07-24

**Authors:** Daniel Shepshelovich, Eitan Amir

**Affiliations:** Eitan Amir: Division of Medical Oncology & Hematology, Department of Medicine, Princess Margaret Cancer Centre and the University of Toronto, Toronto, Ontario, Canada

**Keywords:** randomized controlled trials, cancer, expedited approval, FDA, safety

In order to speed up the availability of new drugs for diseases with unmet need, regulators around the world have created rapid review and approval pathways. For the US Food and Drug Administration (FDA), Fast Track, Priority Review and Breakthrough and Orphan Drug designations can allow for softening of evidentiary standards with rapid review and accelerated approval of some drugs without randomized data [1, 2]. Randomized control trials (RCTs) have been the standard tool for the assessment of the safety and efficacy of new drugs for more than fifty years. Avoiding the need to perform RCTs substantially shortens the time to regulatory approval, as demonstrated by the abbreviated development timelines of crizotinib and pembrolizumab [3, 4]. The post-marketing implications of such strategies have been unknown. In a recent study, new cancer drug indications approved without supporting RCTs were significantly more likely to require post-marketing label modifications for common adverse effects, and also had a higher prevalence of major modifications in warnings and precautions which approached, but did not meet statistical significance [5]. Non-randomized trials included significantly smaller patient sample sizes, and all used surrogate endpoints as their primary outcomes. Additionally, applications not supported by a RCT were more likely to receive accelerated approval and breakthrough therapy designation.

Expedited regulatory pathways have been associated with shorter time to market but also a higher prevalence of post-marketing safety related label modifications in a cohort of cancer and non-cancer drug approvals [6]. Recent data show that 43% of new cancer drugs approved in recent years received breakthrough therapy designation, and that these drugs were associated with faster times to approval despite comparable efficacy to non-breakthrough drugs [7]. Taken as a single body of evidence, faster review and approval processes result in less knowledge on safety and a higher prevalence of post-marketing label changes, without substantially improved efficacy. Additionally, earlier review and approval shifts the process of drug evaluation from clinical trials into routine clinical practice where there is less rigorous collection of patient outcomes. This emphasizes the need for robust pharmacovigilance programs. Healthcare professionals should be aware of this and practice increased vigilance when using such drugs in the early post-approval setting. Patients should be informed of the higher risk involved with these drugs, and regulators should institute appropriate pharmacovigilance and risk management programs to identify emerging adverse events quickly.

An additional consideration of cancer drug approval without supporting RCTs is the quality of the data which forms the basis for clinical decision making as well as for treatment guidelines. The combination of small sample size, lack of control group, surrogate endpoints and expedited approval pathways is concerning and results in uncertainty about whether data derived from non-randomized registration trials is clinically meaningful. While this might be appropriate for urgently needed drugs, the increasing prevalence of drugs approved through expedited regulatory pathways is noteworthy, perhaps driven by industry eager to approve drugs quickly and reduce costs of drug development [8]. Many of these approvals are conditional upon completion of post-approval studies to complement data from the smaller pre-approval trials. Data show that among drugs approved using the FDA’s accelerated approval pathway which have completed post-marketing studies, the majority have efficacy confirmed [9]. Unfortunately, post-approval trials are often delayed for many years, and many share design flaws with the pre-approval trials [9, 10].

**Figure 1:**
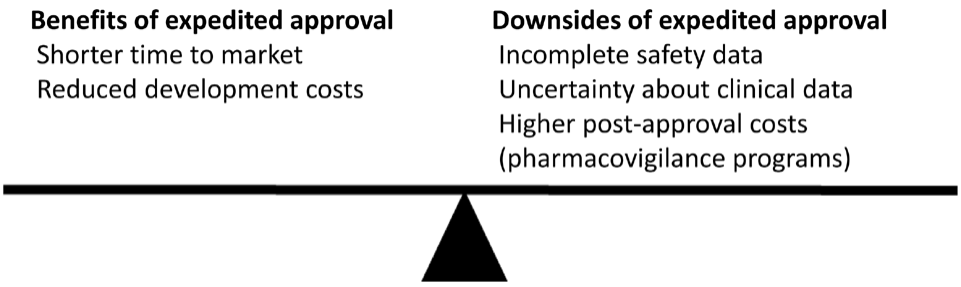
Benefits and downsides of expedited drug approval

There is an inherent trade-off between pre-approval scrutiny of the safety and efficacy of new drugs, and the desire to get potent new drugs to patients without delay (Figure 1). Achieving the optimal balance is challenging. It appears that in recent years, regulators have favored expedited review. However, accumulating data show that there are downsides to this choice. Regulators should consider granting fewer expedited approvals and demand higher quality data, to ensure clinicians can make better decisions based on a more robust understanding of the efficacy and toxicity of new cancer treatments.
